# Supplementary material for: CD83 expression characterizes precursor exhausted T cell population
Source: Commun Biol. 2023 Mar 11;6:258. doi: 10.1038/s42003-023-04631-6 (PMC10008643; doi:10.1038/s42003-023-04631-6)
Supplement: Supplementary file 2 — Supplementary Information [file 42003_2023_4631_MOESM2_ESM.pdf]

# Supplemental Information

## CD83 expression characterizes precursor exhausted T cell population

Zhiwen Wu[1], Toshiaki Yoshikawa[1], Satoshi Inoue[1], Yusuke Ito[1], Hitomi Kasuya[1], Takahiro Nakashima[1,2], Haosong Zhang[1,3], Saki Kotaka[4], Waki Hosoda[5], Shiro Suzuki[4], Yuki Kagoya[1,3,6*]

[1]Division of Immune Response, Aichi Cancer Center Research Institute, Nagoya, Japan

[2]Department of Hematology and Oncology, Nagoya City University Graduate School of Medical Sciences, Nagoya, Japan

[3]Division of Cellular Oncology, Department of Cancer Diagnostics and Therapeutics, Nagoya University Graduate School of Medicine, Nagoya, Japan

[4]Department of Gynecologic Oncology, Aichi Cancer Center, Nagoya, Japan

[5]Department of Pathology and Molecular Diagnostics, Aichi Cancer Center, Nagoya, Japan

[6]Division of Tumor Immunology, Institute for Advanced Medical Research, Keio University School of Medicine

*** Corresponding author information:** Yuki Kagoya, MD, PhD

Keio University School of Medicine

35 Shinanomachi, Shinjuku, Tokyo 160-8582, Japan

Phone: +81-3-5843-6177

E-mail: ykagoya@keio.jp

**Supplementary Figures 1-7**

**Supplementary Tables 1-2**

Supplementary Figure 1

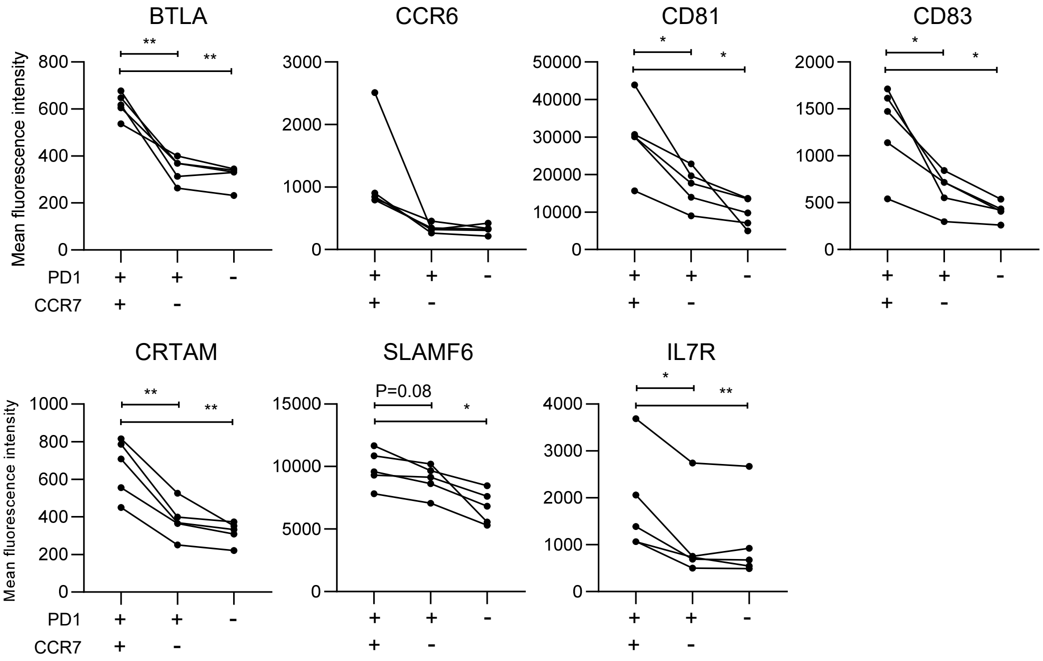


**Supplementary Fig. 1. Phenotypic profiles of precursor and terminally exhausted CAR-T-cells.** Anti-mesothelin or anti-CD19 CAR-T cells were infused into NSG mice engrafted with A375-mesothelin or A375-CD19, and tumor-infiltrating $PD1^{+}CCR7^{+/-}$ or $PD1^{-}$ T cells were analyzed for the indicated surface molecules. The data shown are mean fluorescence intensity of each molecule (n=5 mice, the same samples as shown in Fig. 2d; repeated measures one-way ANOVA with multiple comparison test). * P<0.05, ** P<0.01.

Supplementary Figure 2

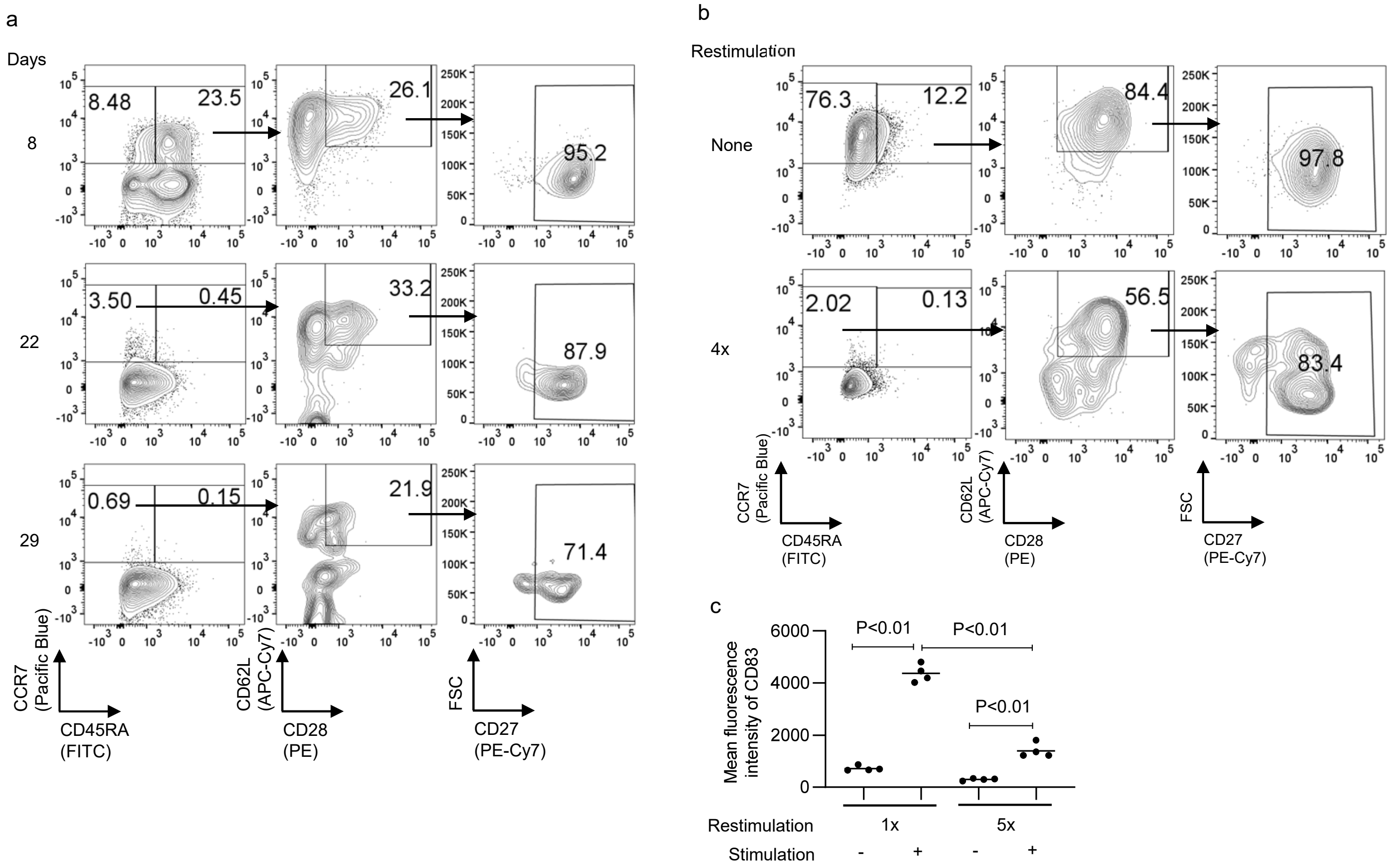


**Supplementary Fig. 2. The difference of CD83 upregulation upon T-cell activation according to the memory differentiation status.** (a) CD19-targeting CAR-T cells were repeatedly stimulated by NALM6 as shown in Figure 4c. Memory markers were analyzed on days 8, 22, and 29. Representative flow cytometry plots of four samples are shown. (b, c) Peripheral blood T cells were weekly stimulated by K562-OKT3/CD80. (b) Representative flow cytometry plots of memory markers analyzed a week after the initial stimulation or after the 4th restimulation. (c) Mean fluorescence intensity of CD83 was analyzed 24 hours after the 1st or 5th restimulation with K562-mOKT3/CD80 (n=4 independent cultures, one-way ANOVA with multiple comparison test). Horizontal lines indicate mean values.

Supplementary Figure 3

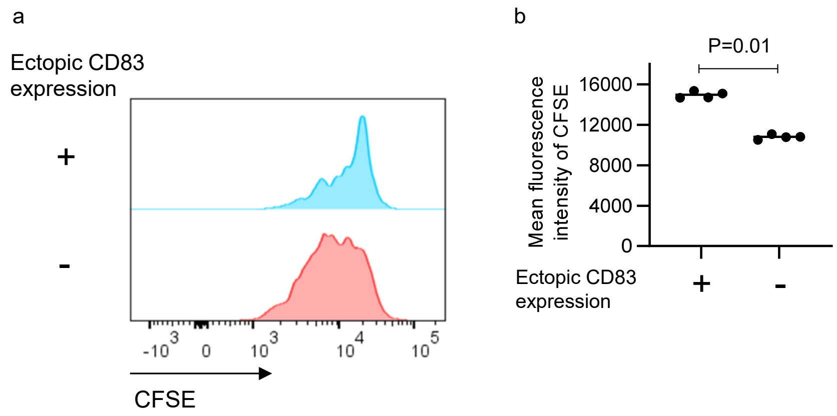


**Supplementary Fig. 3. CD83 attenuates CD8$^{+}$ CAR-T-cell proliferation upon repeated stimulation.** (a, b) Human T cells were transduced with mesothelin-targeting CAR alone or CAR and CD83. The generated CAR-T cells were weekly stimulated by K562-mesothelin. The CAR-T cells were labeled with CFSE before the 4th restimulation and analyzed for cell division. Representative flow cytometry plots (a) and the mean fluorescence intensity of CFSE 5 days after stimulation are shown (b, n=4 independent cultures, unpaired two-tailed *t*-test). Horizontal lines indicate mean values.

Supplementary Figure 4

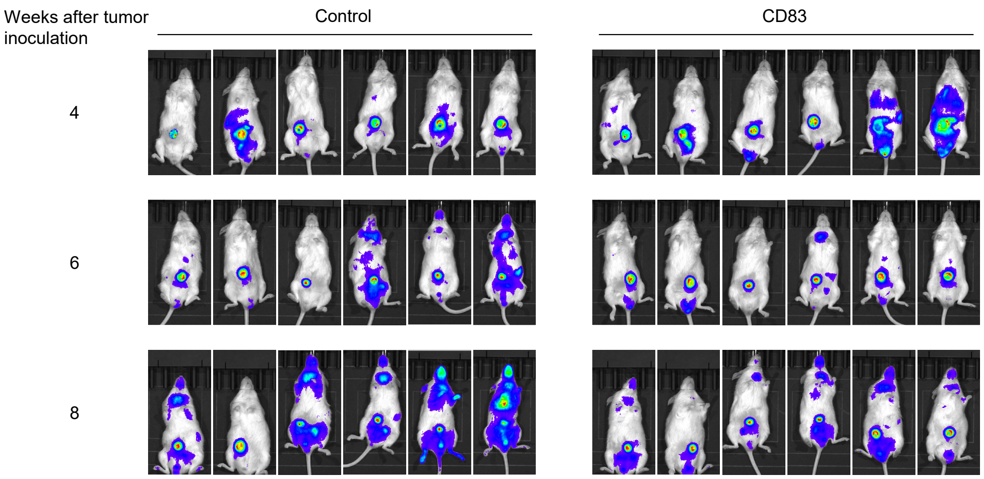


**Supplementary Fig. 4. The effect of CD83 overexpression on *in vivo* antitumor T cell response.** NSG mice inoculated with the mesothelin$^{+}$ cell line AsPC-1 were treated by control or CD83-overexpressing CAR-T cells targeting mesothelin. CAR-T cells were transduced with the luciferase gene and longitudinally monitored for *in vivo* persistence and distribution.

Supplementary Figure 5

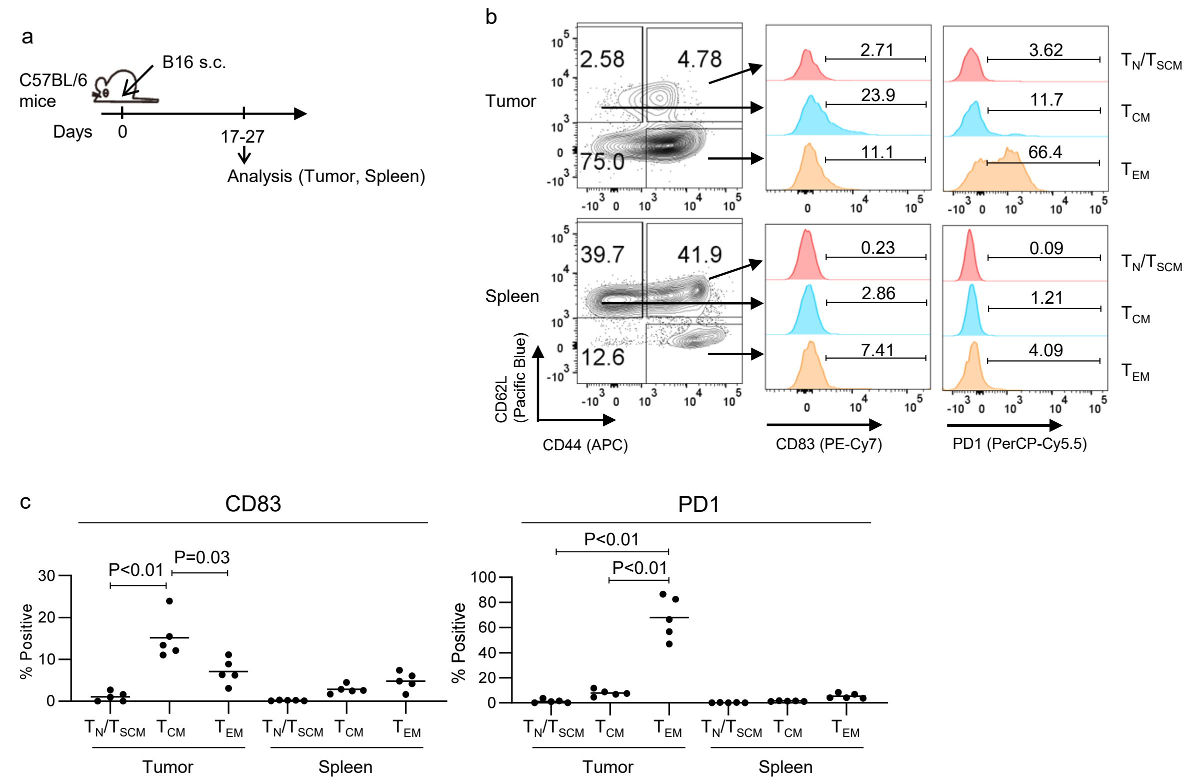


**Supplementary Fig. 5. CD83 expression profiles of tumor-infiltrating T cells in the B16 melanoma model.** (a-c) C57BL/6 mice were subcutaneously inoculated with the melanoma cell line B16. Tumor-infiltrating T cells were analyzed for memory markers as well as CD83 and PD1. (b, c) Representative flow cytometry plots (b) and the frequency of $CD83^+$ and $PD1^+$ T cells at the indicated T-cell subsets within the tumor and spleen (c, n=5 mice, repeated measures one-way ANOVA with multiple comparison test). Horizontal lines indicate mean values.

Supplementary Figure 6

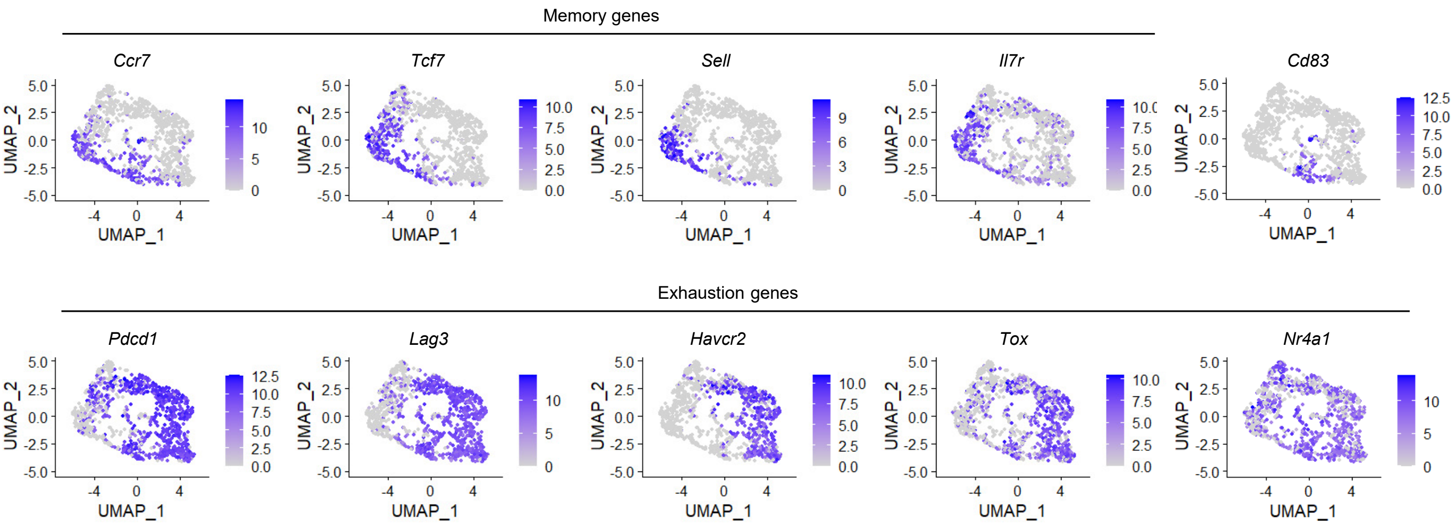


**Supplementary Fig. 6. Expression patterns of CD83 in tumor-infiltrating T cells at the single-cell level.** Single-cell RNA-sequencing data analyzing gene expression profiles of $CD8^+$ T cells within the B16 melanoma tissue were retrieved from GSE86039. Expression levels of the indicated genes were visualized by UMAP.

Supplementary Figure 7

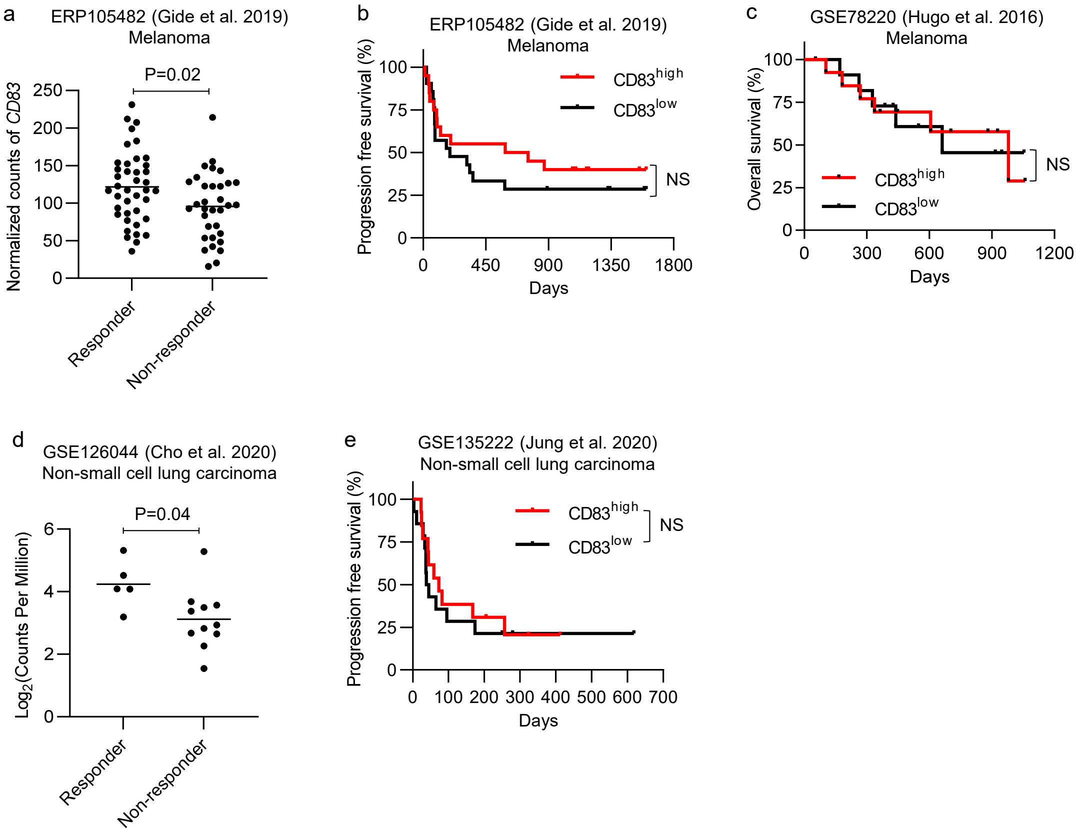


**Supplementary Fig. 7. The prognostic impact of CD83 expression levels in patients treated with immune checkpoint inhibitors.** (a-e) Patients with melanoma (a-c) or lung cancer (d, e) treated with immune checkpoint inhibitors (ICI) were analyzed for the association of CD83 expression levels with prognosis using the indicated datasets. In **a** and **d**, CD83 expression levels were compared between responders and non-responders to ICI (a, n=40 or 33; d, n=5 or 11; two-tailed unpaired *t*-test). Horizontal lines indicate mean values. In **b**, **c**, and **e**, overall survival or progression-free survival was compared between patients grouped according to the expression levels of CD83 (b, n=20 or 21; c, n=12 or 13; e, n=13 or 14; log-rank test). The median expression value of *CD83* in each cohort was used as a cut-off value. NS, not significant.

**Supplementary Table 1. Antibodies used in flow cytometry analysis**

| Name (Fluorochrome-target antigen) | Dilution | Clone | Vendor | Reactivity | Catalog No. |
|---|---|---|---|---|---|
| PE-anti-CD4 | 1:50 | RPA-T4 | BioLegend | human | #300539 |
| APC-anti-CD8 | 1:50 | RPA-T8 | BioLegend | human | #301049 |
| FITC-anti-CD8 | 1:50 | RPA-T8 | BioLegend | human | #301050 |
| APC-R700-anti-CD8 | 1:20 | RPA-T8 | BD Biosciences | human | #565165 |
| BV421-anti-CD8 | 1:50 | RPA-T8 | BioLegend | human | #301036 |
| PE-anti-CD4 | 1:50 | RPA-T4 | BioLegend | human | #300539 |
| FITC-anti-CD45 | 1:50 | HI30 | BioLegend | human | #304038 |
| Alexa 647-anti-CD45 | 1:50 | HI30 | BioLegend | human | #304018 |
| BV711-anti-CD45 | 1:25 | HI30 | BioLegend | human | #304050 |
| PE-Cy7-anti-CD279 (PD1) | 1:16 | EH12.1 | BD Biosciences | human | #561272 |
| Alexa 488-anti-CD279 (PD1) | 1:16 | EH12.2H7 | BioLegend | human | #329936 |
| BV421-anti-CCR7 (CD197) | 1:16 | 3D12 | BD Biosciences | human | #740052 |
| Pacific Blue-anti-CCR7 (CD197) | 1:16 | G043H7 | BioLegend | human | #353210 |
| BB515-anti-CCR7 (CD197) | 1:16 | 3D12 | BD Biosciences | human | #565869 |
| PerCP/Cy5.5-anti-CD271 (NGFR) | 1:50 | C40-1457 | BD Biosciences | human | #560834 |
| PerCP/Cy5.5-anti-CD271 (NGFR) | 1:50 | ME20.4 | BioLegend | human | #345112 |
| Biotin-anti-CD196 (CCR6) | 1:20 | 11A9 | BD Biosciences | human | #559561 |
| Biotin-anti-SLAMF6 (CD352) | 1:20 | REA339 | Miltenyi Biotec | human | #130-105-592 |
| Biotin-anti-CD81 | 1:20 | 5A6 | BioLegend | human | #349514 |
| Biotin-anti-CRTAM (CD355) | 1:20 | REA1225 | Miltenyi Biotec | human | #130-124-099 |
| Biotin-anti-CD272 (BTLA) | 1:20 | MIH26 | Thermo Fisher | human | #13-5979-80 |
| BV421-anti-CD83 | 1:20 | HB15e | BD Biosciences | human | #562630 |
| Alexa 647-anti-CD83 | 1:20 | HB15e | BD Biosciences | human | #305316 |
| Biotin-anti-CD83 | 1:20 | HB15e | BioLegend | human | #305304 |
| BV421-anti-TIM3 (CD366) | 1:16 | 7D3 | BD Biosciences | human | #565562 |
| PE-Cy7-anti-TIM3 (CD366) | 1:16 | F38-2E2 | BioLegend | human | #345014 |
| Biotin-anti-CD127 (IL7Rα) | 1:20 | A019D5 | BioLegend | human | #351346 |
| PE-Streptavidin | 1:100 | NA | Thermo Fisher | NA | #S866 |
| Alexa 647-anti-TCF7 | 1:6 | 7F11A10 | BioLegend | human | #655204 |
| FITC-anti-CD45RA | 1:25 | HI100 | BD Biosciences | human | #555488 |
| APC-Cy7-anti-CD62L | 1:25 | DREG-56 | BioLegend | human | #304814 |
| PE-anti-CD28 | 1:25 | CD28.2 | BioLegend | human | #302908 |
| PE-Cy7-anti-CD27 | 1:25 | M-T271 | BD Biosciences | human | #560609 |
| PE-Cy7-anti-CD27 | 1:25 | M-T271 | BioLegend | human | #356412 |
| PE-anti-Granzyme B | 1:20 | QA16A02 | BioLegend | human | #372208 |
| FITC-anti-Perforin | 1:20 | dG9 | BioLegend | human | #308104 |
| APC-anti-CD107a | 1:100 | H4A3 | BioLegend | human | #328620 |
| FITC-anti-IL2 | 1:15 | MQ1-17H12 | BioLegend | human | #500304 |
| PE-Cy7-anti-IFNγ | 1:10 | 4S.B3 | BioLegend | human | #502528 |
| BV421-anti-TNFα | 1:15 | MAb11 | BioLegend | human | #502932 |
| PE-anti-CD8a | 1:50 | 53-6.7 | BD Biosciences | mouse | #553033 |
| PerCP-Cy5.5-anti-PD1 | 1:20 | 29F.1A12 | BioLegend | mouse | #135208 |
| PE-Cy7-anti-CD83 | 1:16 | Michel-19 | BioLegend | mouse | #121518 |
| Pacific Blue-anti-CD62L | 1:20 | MEL-14 | BioLegend | mouse | #104424 |
| APC-anti-CD44 | 1:50 | IM7 | BioLegend | mouse/human | #103012 |

**Supplementary Table 2. Pathological diagnosis of tumor tissues analyzed in this study.**

| Sample ID | Tumor site (pathological diagnosis) |
|---|---|
| 1 | Ovarian cancer (Endometrioid carcinoma) |
| 2 | Cervical cancer (Squamous cell carcinoma) |
| 3 | Ovarian cancer (Mucinous carcinoma) |
| 4 | Cervical cancer (Adenocarcinoma) |
| 5 | Endmetrial cancer (Endometrioid carcinoma) |
| 6 | Cervical cancer (Small cell neuroendocrine carcinoma) |
| 7 | Cervical cancer (Squamous cell carcinoma) |
| 8 | Cervical cancer (Gastric-type mucinous caricinoma) |
| 9 | Cervical cancer (Squamous cell carcinoma) |
| 10 | Ovarian cancer (Endometrioid carcinoma) |
