# Supplementary material for: CD83 expression characterizes precursor exhausted T cell population
Source: Commun Biol. 2023 Mar 11;6:258. doi: 10.1038/s42003-023-04631-6 (PMC10008643; doi:10.1038/s42003-023-04631-6)
Supplement: Supplementary file 3 — Description of Additional Supplementary Files [file 42003_2023_4631_MOESM3_ESM.pdf]

Description of Additional Supplementary Files

File name: Supplementary Data 1
Description: The source data underlying all the graphs in the paper.
