# Supplementary material for: CD83 expression characterizes precursor exhausted T cell population
Source: Commun Biol. 2023 Mar 11;6:258. doi: 10.1038/s42003-023-04631-6 (PMC10008643; doi:10.1038/s42003-023-04631-6)
Supplement: Supplementary file 5 — Reporting Summary [file 42003_2023_4631_MOESM5_ESM.pdf]

nature portfolio

Corresponding author(s): Yuki Kagoya
Last updated by author(s): Feb 10, 2023

Reporting Summary

Nature Portfolio wishes to improve the reproducibility of the work that we publish. This form provides structure for consistency and transparency in reporting. For further information on Nature Portfolio policies, see our Editorial Policies and the Editorial Policy Checklist.

Statistics

For all statistical analyses, confirm that the following items are present in the figure legend, table legend, main text, or Methods section.

n/a	Confirmed	
☐	☒	The exact sample size (n) for each experimental group/condition, given as a discrete number and unit of measurement
☐	☒	A statement on whether measurements were taken from distinct samples or whether the same sample was measured repeatedly
☐	☒	The statistical test(s) used AND whether they are one- or two-sided<br>*Only common tests should be described solely by name; describe more complex techniques in the Methods section.*
☒	☐	A description of all covariates tested
☐	☒	A description of any assumptions or corrections, such as tests of normality and adjustment for multiple comparisons
☐	☒	A full description of the statistical parameters including central tendency (e.g. means) or other basic estimates (e.g. regression coefficient) AND variation (e.g. standard deviation) or associated estimates of uncertainty (e.g. confidence intervals)
☐	☒	For null hypothesis testing, the test statistic (e.g. F, t, r) with confidence intervals, effect sizes, degrees of freedom and P value noted<br>*Give P values as exact values whenever suitable.*
☒	☐	For Bayesian analysis, information on the choice of priors and Markov chain Monte Carlo settings
☒	☐	For hierarchical and complex designs, identification of the appropriate level for tests and full reporting of outcomes
☐	☒	Estimates of effect sizes (e.g. Cohen's d, Pearson's r), indicating how they were calculated

Our web collection on statistics for biologists contains articles on many of the points above.

Software and code

Policy information about availability of computer code

Data collection	NA
Data analysis	GraphPad Prism version 9.5.0 for statistical analysis; FlowJo (version 10.8.1) for flow cytometry data analysis; Hisat2 (version 2.1.0), HTseq (version 0.11.2), fastp (version 0.20.0), samtools (version 1.9), edgeR (version 3.32.0), and R (version 4.0.5) for RNA-seq data analysis; Seurat (version 4.2.0) and singleseqgset (version 0.1.2.9000) for single-cell RNA-seq analysis.

For manuscripts utilizing custom algorithms or software that are central to the research but not yet described in published literature, software must be made available to editors and reviewers. We strongly encourage code deposition in a community repository (e.g. GitHub). See the Nature Portfolio guidelines for submitting code & software for further information.

Data

Policy information about availability of data

All manuscripts must include a data availability statement. This statement should provide the following information, where applicable:

- Accession codes, unique identifiers, or web links for publicly available datasets
- A description of any restrictions on data availability
- For clinical datasets or third party data, please ensure that the statement adheres to our policy

The raw data used in this study are provided in Supplementary Data 1. Other data or information related to this study are available from the corresponding author (Y. K.) upon reasonable request.

Human research participants

Policy information about studies involving human research participants and Sex and Gender in Research.

Reporting on sex and gender	NA
Population characteristics	Healthy donor-derived PBMCs were purchased from Cellular Technology Limited. Population characteristics of individual donors are not available to researchers.<br>Tumor infiltrating lymphocytes (TIL) samples were obtained from the patients at Aichi Cancer Center (Nagoya, Japan). Pathological diagnosis of tumor tissues is presented in Supplementary Table 2.
Recruitment	TIL samples were obtained from the patients who consented to this study. Written informed consent was obtained from all the patients. There is no potential selection bias.
Ethics oversight	This study was approved by the Research Ethics Board of the Aichi Cancer Center, Nagoya, Japan.

Note that full information on the approval of the study protocol must also be provided in the manuscript.

Field-specific reporting

Please select the one below that is the best fit for your research. If you are not sure, read the appropriate sections before making your selection.

☒ Life sciences ☐ Behavioural & social sciences ☐ Ecological, evolutionary & environmental sciences

For a reference copy of the document with all sections, see nature.com/documents/nr-reporting-summary-flat.pdf

Life sciences study design

All studies must disclose on these points even when the disclosure is negative.

Sample size	Samples sizes were estimated based on preliminary experiments. We did not use a statistical method to determine sample size beforehand.
Data exclusions	No data were excluded throughout the studies.
Replication	All of the presented data were obtained using multiple (at least three) samples. We repeated the same experiments if required and confirmed reproducibility.
Randomization	In mouse tumor treatment models, mice were equally and randomly allocated to two groups.
Blinding	The investigators were not blinded for sample identity during data collection. Our analysis is based on the objectively measurable data such as absolute cell counts, frequency, and mean fluorescence intensity. Sample blinding does not affect analysis of these data.

Reporting for specific materials, systems and methods

We require information from authors about some types of materials, experimental systems and methods used in many studies. Here, indicate whether each material, system or method listed is relevant to your study. If you are not sure if a list item applies to your research, read the appropriate section before selecting a response.

Materials & experimental systems

n/a	Involved in the study	
☐	☒	Antibodies
☐	☒	Eukaryotic cell lines
☒	☐	Palaeontology and archaeology
☐	☒	Animals and other organisms
☐	☒	Clinical data
☒	☐	Dual use research of concern

Methods

n/a	Involved in the study	
☒	☐	ChIP-seq
☐	☒	Flow cytometry
☒	☐	MRI-based neuroimaging

Antibodies

Antibodies used	All of the antibodies with required information were listed in Supplementary Table 1.

Validation	We confirmed that all the antibodies used in this study are validated for use in each of the specific experiments by the manufacturer. Validation data are available on the manufacturers' websites.

Eukaryotic cell lines

Policy information about cell lines and Sex and Gender in Research

Cell line source(s)	The erythroleukemia cell line K562 and the mouse melanoma cell line B16 were purchased from the Japanese Collection of Research Bioresources cell bank (Osaka, Japan). The CD19+ B-cell leukemia cell line NALM6 and the mouse colon carcinoma cell line Colon-26 were obtained from the Cell Resource Center for Biomedical Research, Tohoku University (Sendai, Japan). The A375 melanoma, PG13 retroviral packaging cell line, and AsPC1 pancreatic cancer cell line were obtained from the American Type Culture Collection (Manassas, VA, USA). The Plat-A and Plat-E packaging cell lines were kindly provided by Dr. T. Kitamura (University of Tokyo, Tokyo, Japan).
Authentication	Cell line authentication was performed by the ATCC, JCRB and Cell Resource Center for Biomedical Research using the STR-based technologies. All the cell lines were directly purchased from these cell banks. Cell line authentication was not carried out by the authors.
Mycoplasma contamination	Mycoplasma testing was performed by the cell banks before shipping. It was not repeated in our laboratory.
Commonly misidentified lines (See ICLAC register)	None of the cell lines used are listed in the ICLAC.

Animals and other research organisms

Policy information about studies involving animals; ARRIVE guidelines recommended for reporting animal research, and Sex and Gender in Research

Laboratory animals	We used 4- to 10-week-old male NSG mice (The Jackson Laboratories) and 4- to 10-week-old female BALB/c and C57BL/6 mice (The Jackson Laboratories).
Wild animals	This study did not involve wild animals.
Reporting on sex	Our findings are not affected by the sex of the animals.
Field-collected samples	This study did not involve sample collection from the field.
Ethics oversight	All animal experiments were approved by the Animal Care and Use Committee of Aichi Cancer Center Research Institute (Nagoya, Japan).

Note that full information on the approval of the study protocol must also be provided in the manuscript.

Clinical data

Policy information about clinical studies
All manuscripts should comply with the ICMJE guidelines for publication of clinical research and a completed CONSORT checklist must be included with all submissions.

Clinical trial registration	Not applicable
Study protocol	Not applicable
Data collection	Surgically resected tumor specimens derived from patients with ovarian, endometrial, and cervical cancer were obtained after written informed consent.
Outcomes	Not applicable

Flow Cytometry

Plots

Confirm that:

- [x] The axis labels state the marker and fluorochrome used (e.g. CD4-FITC).
- [x] The axis scales are clearly visible. Include numbers along axes only for bottom left plot of group (a 'group' is an analysis of identical markers).
- [x] All plots are contour plots with outliers or pseudocolor plots.
- [x] A numerical value for number of cells or percentage (with statistics) is provided.

Methodology

Sample preparation	Human T cells are derived from healthy donors-derived peripheral blood mononuclear cells or tumor-infiltrating lymphocytes (TILs) obtained from primary or metastatic tumor. TIL samples were dissociated into single-cell suspensions using the gentleMACS Dissociator (Miltenyi Biotech). In mouse experiments, tumor samples and spleen cells were grinded with a syringe and passed through a 70 um filter or dissociated using the gentleMACS Dissociator.
Instrument	LSRFortessa and FACSCAriaIII (BD Biosciences)
Software	Flowjo software (version 10.8.1) (BD Biosciences)
Cell population abundance	The purity was not examined after sorting due to the low number of collected cells.
Gating strategy	After gating on forward scatter (FSC) and side scatter (SSC), doublets were excluded by FSC-H vs. FSC-W as well as SSC-H vs. SSC-W gating. To define a population expressing a specific marker, gating was determined based on the positive or negative control samples. We described how gating threshold was determined in each Figure legend when necessary.

☒ Tick this box to confirm that a figure exemplifying the gating strategy is provided in the Supplementary Information.
